# The Use of Mobile Personal Health Records for Hemoglobin A1c Regulation in Patients With Diabetes: Retrospective Observational Study

**DOI:** 10.2196/15372

**Published:** 2020-06-02

**Authors:** Dongjin Seo, Yu Rang Park, Yura Lee, Ji Young Kim, Joong-Yeol Park, Jae-Ho Lee

**Affiliations:** 1 Department of Medicine Yonsei University College of Medicine Seoul Republic of Korea; 2 Department of Biomedical Systems Informatics Yonsei University College of Medicine Seoul Republic of Korea; 3 Department of Information Medicine Asan Medical Center, University of Ulsan College of Medicine Seoul Republic of Korea; 4 Medical Information Office Asan Medical Center Seoul Republic of Korea; 5 Department of Endocrinology and Metabolism Asan Medical Center University of Ulsan College of Medicine Seoul Republic of Korea; 6 Department of Emergency Medicine Asan Medical Center University of Ulsan College of Medicine Seoul Republic of Korea

**Keywords:** personal health record, mobile health, electronic medical record, diabetes mellitus, glycated hemoglobin A

## Abstract

**Background:**

The effectiveness of personal health records (PHRs) in diabetes management has already been verified in several clinical trials; however, evidence of their effectiveness in real-world scenarios is also necessary. To provide solid real-world evidence, an analysis that is more accurate than the analyses solely based on patient-generated health data should be conducted.

**Objective:**

This study aimed to conduct a more accurate analysis of the effectiveness of using PHRs within electronic medical records (EMRs). The results of this study will provide precise real-world evidence of PHRs as a feasible diabetes management tool.

**Methods:**

We collected log data of the *sugar* function in the My Chart in My Hand version 2.0 (MCMH 2.0) app from Asan Medical Center (AMC), Seoul, Republic of Korea, between December 2015 and April 2018. The EMR data of MCMH 2.0 users from AMC were collected and integrated with the PHR data. We classified users according to whether they were continuous app users. We analyzed and compared their characteristics, patterns of hemoglobin A_1c_ (HbA_1c_) levels, and the proportion of successful HbA_1c_ control. The following confounders were adjusted for HbA_1c_ pattern analysis and HbA_1c_ regulation proportion comparison: age, sex, first HbA_1c_ measurement, diabetes complications severity index score, sugar function data generation weeks, HbA_1c_ measurement weeks before MCMH 2.0 start, and generated sugar function data count.

**Results:**

The total number of MCMH 2.0 users was 64,932, with 7453 users having appropriate PHRs and diabetes criteria. The number of continuous and noncontinuous users was 133 and 7320, respectively. Compared with noncontinuous users, continuous users were younger (*P*<.001) and had a higher male proportion (*P*<.001). Furthermore, continuous users had more frequent HbA_1c_ measurements (*P*=.007), shorter HbA_1c_ measurement days (*P*=.04), and a shorter period between the first HbA_1c_ measurement and MCMH 2.0 start (*P*<.001). Diabetes severity–related factors were not statistically significantly different between the two groups. Continuous users had a higher decrease in HbA_1c_ (*P*=.02) and a higher proportion of regulation of HbA_1c_ levels to the target level (*P*=.01). After adjusting the confounders, continuous users had more decline in HbA_1c_ levels than noncontinuous users (*P*=.047). Of the users who had a first HbA_1c_ measurement higher than 6.5% (111 continuous users and 5716 noncontinuous users), continuous users had better regulation of HbA_1c_ levels with regard to the target level, 6.5%, which was statistically significant (*P*=.04).

**Conclusions:**

By integrating and analyzing patient- and clinically generated data, we demonstrated that the continuous use of PHRs improved diabetes management outcomes. In addition, the HbA_1c_ reduction pattern was prominent in the PHR continuous user group. Although the continued use of PHRs has proven to be effective in managing diabetes, further evaluation of its effectiveness for various diseases and a study on PHR adherence are also required.

## Introduction

### Background

Diabetes mellitus is a global issue, and its contribution to numerous complications and increased mortality is well known. Moreover, diabetes prevalence is constantly growing, a trend that might continue until 2030 or longer [1,2]. According to the American Diabetes Association (ADA), diabetes care is mainly based on insulin delivery [3]. According to the Korean Diabetes Association (KDA), the target value of hemoglobin A_1c_ (HbA_1c_) is recommended to be 6.5% for patients with type 2 diabetes, and antihyperglycemic therapy is mainly considered in Korea. Metformin is considered to be the first-line therapy. However, these traditional drug therapies result in inevitable hypoglycemic events and body weight change. An unachieved glycemic target can only be solved by increasing drugs in mono, dual, or triple therapy [4]. Traditional methods are expensive, and this is becoming a national health care problem [5,6]. To overcome several limitations of traditional diabetes management, mobile health (mHealth) technology and personal health record (PHR) implementation have been suggested as innovative solutions.

In the diabetes management market, new treatments with new devices and apps are being introduced. Most functions of diabetes apps focus on maintaining a blood glucose diary. Some are also connected with blood glucose sensors and treatment devices. Among diabetes apps, *OneTouch Reveal* had the best validation [7]. This app is wirelessly connected to the *OneTouch Verio Flex meter,* making users self-monitor their blood glucose. Blood glucose data are delivered to health care professionals, and users receive text message feedback [8]. Technologies using automatic alarm systems have also been introduced. The Dexcom G6 Continuous Glucose Monitoring system effectively reduced hyperglycemia and also hypoglycemic events with the *Urgent Low Soon* automatic alert system [9]. Monitoring insulin delivery became possible with internet-based connections. *NovoPen 6* and *NovoPen Echo Plus* are called *smart insulin pens*, which can monitor the insulin injection amount and provide both health providers and patients treatment accuracy [10,11].

Previous studies have shown the health improvement of PHR users, thus suggesting that a digital health care system is feasible for improving health behavior and chronic conditions. According to a systematic review, users experienced a positive effect on their health-related behavior and clinical results when using health apps on their mobile devices [12]. Another systematic review in South Korea showed that mHealth interventions were effective in improving self-management behaviors, biomarkers, or patient-reported outcome measures [13]. However, the positive effect of mHealth and PHR interventions is not always ensured.

In diabetes care, PHR and mHealth interventions are expected to be effective treatments. WellDoc, a remote blood glucose monitoring system, was effective in lowering HbA_1c_ levels, thereby improving clinical, behavioral, and diabetes knowledge outcomes [14]. A phone-based treatment and behavioral coaching intervention also improved HbA_1c_ levels [15]. A similar improvement in HbA_1c_ control for type 2 diabetes was seen with another mobile-based intervention [16]. The addition of a tailored mobile coaching system for patients with diabetes showed reduced HbA_1c_ levels and improved diabetes self-management; the results were reproducible and durable [17].

Along with the expectations of the clinical implications of PHRs, some concerns and slightly controversial results have been reported. Despite its advantages, studies have reported the barriers in PHR implementation. Patients are concerned about the security of their health information. Health care providers are concerned about patients altering their own PHR information. Other issues are that there is no practical difference in health outcomes, the use of stand-alone PHRs with electronic medical records (EMRs) and electronic health records, and a low health care literacy rate, which can diminish the benefits of PHRs [18]. Moreover, the barriers associated with patients’ age, sex, socioeconomic status, education level, internet and computer access, and health have been reviewed [19]. Contrasting results of the relation between PHR use and diabetes management have been reported. A study using a regression model claimed that there was no association between the increasing number of days of PHR use and better diabetes quality measure profiles [20].

### Objectives

In this study, we used a 4-year mobile PHR (mPHR) log and users’ EMR data to analyze the effects of diabetes management on the continuous use of the PHR system distributed by a tertiary hospital in South Korea. A study with the earlier version of the mPHR app was conducted to verify characteristics of continuous users [21], and patient-generated health data (PGHD) of continuous users had a higher proportion of a chronic disease diagnosis, such as diabetes, than noncontinuous users [22]. With the new version, we will verify its effect in glycemic control on patients with diabetes. To the best of our knowledge, this is the first study to verify the effectiveness of disease management by integrating a long-term mPHR log and EMR data.

## Methods

### Data and Mobile Personal Health Record Description

**Figure 1:**
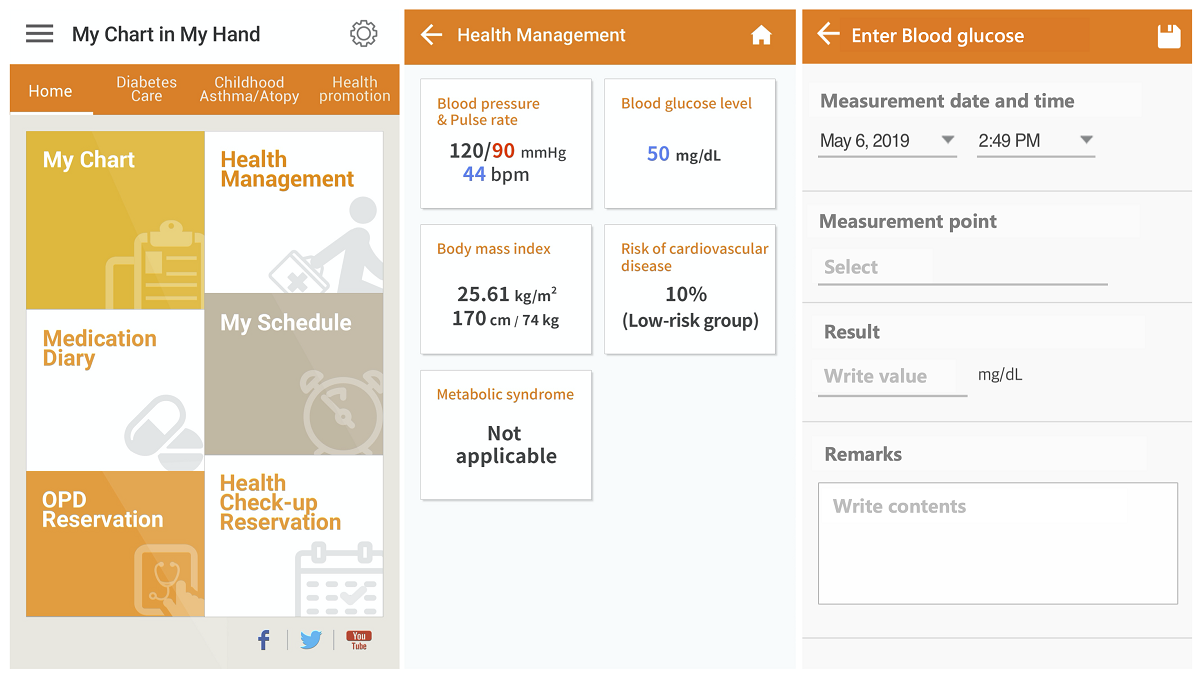
Screenshots of My Chart in My Hand version 2.0. Inputting data in the sugar function follows from the home page to Enter Blood Glucose.

We collected log data from an mPHR app called My Chart in My Hand (MCMH) and their EMR data at the Asan Medical Center (AMC), which is the largest general hospital in South Korea. Launched in January 2011, MCMH is the first mPHR in South Korea; it enables patients to view and manage their own health records [21]. We used the MCMH version 1.0 log to identify patterns of continuous generation of PGHD in specific populations [22]. This study performed a diabetes management analysis using the MCMH version 2.0 log and EMR data. For patients with diabetes, MCMH version 2.0 provides *sugar*, *diabetes calendar*, *insulin* *treatment*, *food intake*, and *exercise* input functions. Among these functions, we only used the log data of the *sugar* and *diabetes calendar* function; the remaining functions had very few records. The items in Figure 1 show the details of the *sugar* function. Users enter the date, time, situation, and result of their blood glucose measurement in these PGHD functions.

We also gathered demographic and medical record information of patients, such as age, sex, residence, and health information, including hospital visits, HbA_1c_ level, diagnosis, and medication data, using our clinical research data warehouse.

### Study Design

MCMH version 2.0 replaced MCMH version 1.0 on December 31, 2015, but some patients had already created their accounts in December 2015 before the replacement. For each user, the records generated in MCMH version 2.0 functions were analyzed, but only records generated after account creation were used.

The user log of the *sugar* function contained user access ID and time stamps of data input. We gathered the HbA_1c_ measurement results of MCMH version 2.0 users from January 2014 to November 2018.

For user selection, we used the criteria of diabetes for diagnosis. First, the criterion of Glasheen et al [23] was adopted: a user should have one or more International Classification of Diseases 10th Revision (ICD-10) diabetes codes in the diagnosis record, which are E08, E09, E10, E11, and E13. Second, the HbA_1c_ cutoff value of 6.5% for diagnosing diabetes was used [24]. For the complication classification and diabetes complications severity index (DCSI) scoring, the selected complication fields from the diagnosis record were retinopathy, nephropathy, neuropathy, cerebrovascular, cardiovascular, peripheral vascular disease, and metabolic complications. DCSI scoring used the criteria of the study by Glasheen et al [23]. However, urine laboratory data were not included in DCSI scoring because of its unavailability. Above all, we classified all diseases according to ICD-10.

The criterion for whether a user was a continuous user was adopted from the PGHD pattern analysis study of MCMH version 1.0: a user entering data in the *sugar* function at least once per week and doing so for at least four weeks (28 days) [22].

We analyzed the pattern of HbA_1c_ levels with the trend line slope of HbA_1c_ levels. The fluctuation of HbA_1c_ levels was compared with the *r*-squared value of the trend line and the standard deviation of the patient’s HbA_1c_ level.

In this study, the trend line slope considerably depended on the measurement days between the first and last HbA_1c_ measurement. Therefore, we created a patient filter called *appropriate HbA_1c_ measurement*. This criterion excluded patients with short periods between measures because a short period will lead to an exaggeratedly steep slope, which is inappropriate for the analysis. The criterion for an appropriate HbA_1c_ measurement is patients should have at least two HbA_1c_ measurements and the period between the first and last HbA_1c_ measurement should be over 100 days. To normalize the effect of measurement days between the first and last HbA_1c_ measurement, we defined a variable called *decline*. *Decline* is defined as a trend line slope times the period (in days) divided by 100. This normalization is represented in the equation in Multimedia Appendix 1.

This study was approved by the Institutional Review Board (IRB) of the AMC (IRB number: 2018-0321). The need for informed consent was waived by the ethics committee because this study utilized routinely collected log data that were anonymously managed at all stages, including during data cleaning and statistical analyses.

### Study Participants

**Figure 2:**
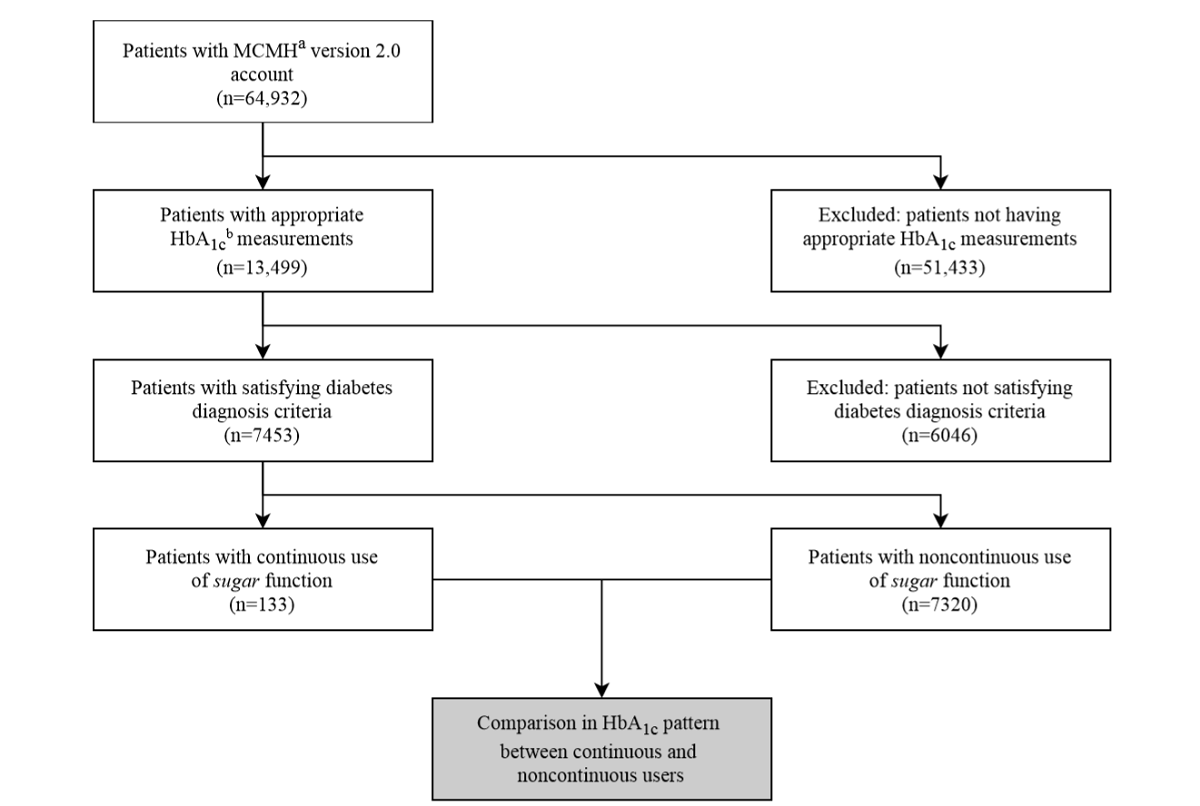
Patient inclusion and exclusion criteria (white boxes) and flow through the study. The gray box shows user hemoglobin A_1c_ (HbA_1c_) analyses. Criteria for appropriate HbA_1c_ measurement: two or more HbA_1c_ measurements, duration of the first and last measurement over 100 days, and creating My Chart in My Hand version 2.0 account during HbA_1c_ measurement. Criteria for diabetes diagnosis: having International Classification of Diseases 10th Revision code E08, E09, E10, E11, or E13 or first HbA_1c_ measurement ≥6.5%. Criteria for continuous use of sugar function: patient-generated health data entered in the sugar function at least once per week and used for at least 28 days. ^a^HbA_1c_: hemoglobin A_1c_; ^b^MCMH: My Chart in My Hand.

Figure 2 shows the patient selection flow in this study. Among 64,932 users who downloaded and created an MCMH version 2.0 account, we first excluded 51,433 users with inappropriate HbA_1c_ measurements. We considered 13,499 users with the appropriate HbA_1c_ measurements, excluded 6046 users without diabetes, and selected 7453 users with diabetes.

### Data Analysis

We first compared the general characteristics of continuous (n=133) and noncontinuous users (n=7320). The following characteristics were compared: age, gender proportion, *sugar* and *diabetes calendar* function use pattern, HbA_1c_ measurement pattern, HbA_1c_ value, DCSI score, and complication proportion. A Student *t* test was conducted for the comparison of age, the number of HbA_1c_ measurements, measurement days, and measurement days before MCMH version 2.0 start. A Wilcoxon rank-sum test was used for individual *sugar* and *diabetes calendar* function data generation, HbA_1c_ measure frequency, first HbA_1c_ measurement, and DCSI score comparison. The median test was used for the individual *sugar* and *diabetes* *calendar* function data generation comparison. The Z test was conducted for *sugar* and *diabetes* function generation user proportion, first HbA_1c_ measurement over 6.5% proportion, and complications proportion comparisons. For gender proportion comparison and DCSI score distribution, a chi-square test was used.

Next, comparative analyses of HbA_1c_ *decline*, *r*-squared value, and standard deviation between continuous and noncontinuous users were performed. We used the Shapiro-Wilk test and D’Agostino K-squared test to determine if these data followed a normal distribution. HbA_1c_ *decline*, *r*-squared value, and standard deviation were compared using the Wilcoxon rank-sum test. For confounder adjustment, we used an analysis of covariance (ANCOVA) with some variables: continuous use, age, sex, first HbA_1c_ measurement, DCSI, *sugar* function data generation weeks, HbA_1c_ measurement in weeks before MCMH version 2.0 start, and *sugar* function data generation count.

Finally, the Z test was conducted for comparing the proportions of 4 groups between continuous and noncontinuous users. The 4 groups were divided by whether the first HbA_1c_ measurement was higher or lower than 6.5% and whether the last HbA_1c_ measurement was higher or lower than 6.5%. For confounder adjustment, multivariable logistic regression was used for users with the first HbA_1c_ measurement over 6.5%. The same variables, as used in ANCOVA, were used for logistic regression. Data analyses were conducted using *Python* 3.6.7, with *Jupyter Notebook*.

## Results

### Overall Characteristics

Within 29 months of operation of MCMH version 2.0, 64,932 users created an account and logged in at least once. Among these users, 7453 users were selected on the basis of the inclusion criteria of this study. Approximately 1.78% (133/7453) of these users were continuous users, and 98.22% (7320/7453) were noncontinuous users. Continuous and noncontinuous users had no statistically significant difference in the number of HbA_1c_ measurements and the period between the first and last HbA_1c_ measurements.

Table 1 summarizes the results of a basic characteristic analysis between continuous and noncontinuous users. In Table 1, measure frequency refers to the number of measurements per day, measurement days refers to days between the first and last HbA_1c_ measurement, and measurement days before MCMH version 2.0 start refers to days between the first HbA_1c_ measurement and MCMH version 2.0 account generation period. Compared with noncontinuous users, continuous users were younger (mean 53.59, SD 9.89 years vs mean 57.58, SD 11.95 years, respectively) and had a higher male proportion (110/133, 82.7% vs 4859/7320, 66.38%, respectively), which was statistically significant (both *P*<.001). The number of HbA_1c_ measurements was not significantly different. The frequency and period between the first and last measurements exhibited a significant difference between continuous and noncontinuous users (*P*=.007 and *P*=.04, respectively). The proportion of patients with the first HbA_1c_ measurement below 6.5% had no significant difference (*P*=.14), but continuous users had a higher first HbA_1c_ measurement, and this was statistically significant (*P*=.01). Furthermore, among continuous users, there were a higher proportion of users who generated data in the sugar function and diabetes calendar function (both *P*<.001). Continuous users also entered more sugar and diabetes calendar data (both *P*<.001). The DCSI score had no significant difference (*P*=.99). The proportion of complications, defined by the DCSI criteria, also showed no significant difference between continuous and noncontinuous users. Although the difference was statistically insignificant, retinopathy and cardiovascular complications had a proportional difference.

The DCSI score proportion of continuous and noncontinuous users had no significant difference in the chi-square test. This can be found in Multimedia Appendix 2. Among the 14 DCSI scores, those with zero proportion in both patient groups (scores 10, 12, and 13) were excluded in the analysis using the chi-square test, because calculation with the chi-square test is only possible when each score does not have zero proportion in any group.

**Table 1:** General characteristics of continuous and noncontinuous users.

Variables			Users		Total (N=7453)	*P* value^a^	
			Continuous (n=133)	Noncontinuous (n=7320)			
Age (years), mean (SD)			53.59 (9.89)	57.58 (11.95)	57.51 (11.92)	<.001	
**Sex, n (%)**							<.001
	Male		110 (82.7)	4859 (66.37)	4969 (66.67)		
	Female		23 (17.3)	2461 (33.62)	2484 (33.33)		
**Sugar function**							
	Data generated by users, n (%)		133 (100.0)	289 (3.95)	422 (5.66)	<.001	
	Total data generated, n		22,350	1345	23,695	—^b^	
	**Individually generated data**					<.001	
		Mean (SD)	168.0 (204.0)	0.2 (1.8)	3.2 (35.1)		
		Median (IQR)	97 (43-186)	0 (0-0)	0 (0-0)		
**Diabetes calendar function**							
	Data generated by users, n (%)		133 (100.0)	297 (4.06)	430 (5.77)	<.001	
	Total data generated, n		16,407	1453	17,860	—	
	**Individually generated data**					<.001	
		Mean (SD)	123.4 (143.3)	0.2 (4.0)	2.4 (25.4)		
		Median (IQR)	67 (35-145)	0 (0-0)	0 (0-0)		
**HbA_1c_^c^** **, mean (SD)**							
	Number of measurements		12.44 (6.90)	11.90 (6.82)	11.92 (6.82)	.38	
	Measure frequency		0.011 (0.010)	0.009 (0.005)	0.009 (0.005)	.007	
	Measurement days		1254 (461)	1336 (445)	1335 (446)	.04	
	Measurement days before MCMH^d^ version 2.0 start		546 (348)	712 (377)	710 (377)	<.001	
First HbA_1c_ measurement ≥6.5%, n (%)			111 (83.4)	5716 (78.09)	5827 (78.18)	.14	
First HbA_1c_ measurement, mean (SD)			7.86 (1.78)	7.51 (1.62)	7.51 (1.62)	.01	
DCSI^e^, mean (SD)			1.17 (1.65)	1.15 (1.64)	1.15 (1.64)	.99	
**Complications, n (%)**							
	Retinopathy or ophthalmic		31 (23.3)	1516 (20.71)	1547 (20.75)	.46	
	Nephropathy		13 (9.8)	765 (10.45)	778 (10.44)	.80	
	Neuropathy		23 (17.3)	1267 (17.31)	1290 (17.31)	>.99	
	Cerebrovascular		20 (15.0)	950 (13.00)	970 (13.01)	.48	
	Cardiovascular		16 (12.0)	1366 (18.7)	1382 (18.54)	.05	
	Peripheral vascular disease		1 (0.8)	59 (0.8)	60 (0.81)	.94	
	Metabolic complications		1 (0.8)	37 (0.5)	38 (0.51)	.69	

^a^Chi-square test or Z test (for categorical variables); Student *t* test or Wilcoxon rank-sum test (for continuous variables).

^b^Statistical comparison was not conducted in total generated data of sugar and diabetes calendar function.

^c^HbA_1c_: hemoglobin A_1c_.

^d^MCMH: My Chart in My Hand.

^e^DCSI: diabetes complications severity index.

### Hemoglobin A_1c_ Pattern Analysis According to Continuous Use

**Figure 3:**
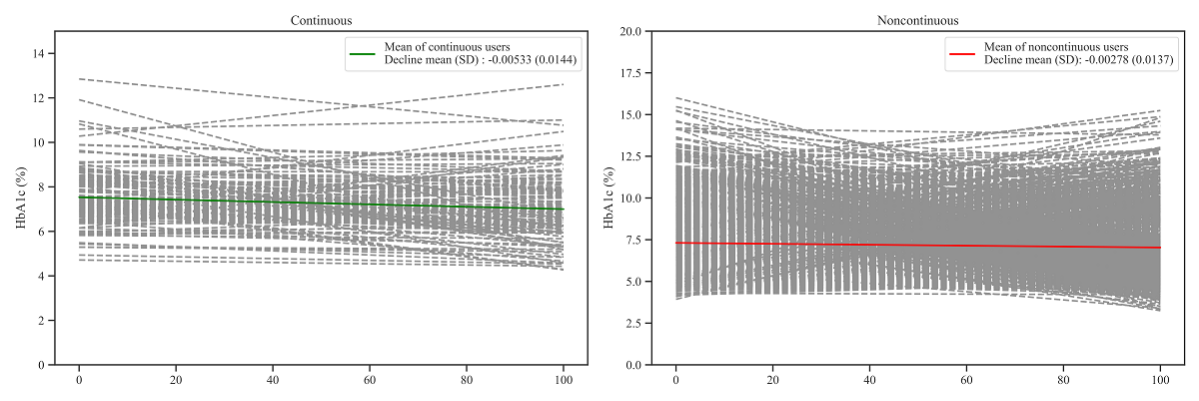
Hemoglobin A_1c_ (HbA_1c_) patterns (decline, r-squared value, and SD) of continuous and noncontinuous users. The x-axis is the percentage of days past from the first HbA_1c_ measurement compared with the period between the first and last HbA_1c_ measurements. The dashed lines are the HbA_1c_ decline of each patient. The slope and y-axis intercept of the continuous lines indicates the mean of slope and y-axis of patients, respectively.

Figure 3 shows the trend of the HbA_1c_ pattern for continuous and noncontinuous users. The HbA_1c_ *decline* of continuous and noncontinuous users was also compared. The HbA_1c_ *decline* (mean −0.00533, SD 0.0144) in continuous users was significantly steeper than that of noncontinuous users (mean −0.00278, SD 0.0137; *P*=.02). The SD of continuous users (mean 0.832, SD 0.574) was significantly higher than that of noncontinuous users (mean 0.719, SD 0.541; *P*=.005). However, the *r*-squared value had no statistically significant difference between continuous and noncontinuous users (*P*=.40).

When adjusting confounders that can contribute to the *decline*, continuous use had a statistically significant effect (*P*=.047) on making *decline* steeper, as seen in Table 2. In addition, age, first HbA_1c_ measurement, DCSI, weeks of *sugar* function data generation, and HbA_1c_ measurement in weeks before MCMH version 2.0 start showed statistically significant effects (*P*=.004; *P*<.001; *P=*.01; *P=*.003; *P*<.001, respectively).

**Table 2:** Results of adjusting confounders with the analysis of covariance in decline comparison.

Variables	*F* test (*df*=1)	*P* value
Continuous users	3.94	.047
Age (years)	8.07	.004
Sex	0.17	.68
First HbA_1c_^a^ measurement	3054.90	<.001
DCSI^b^	6.45	.01
*Sugar* function data generation (weeks)	8.68	.003
HbA_1c_ measurement weeks before MCMH version 2.0 start	154.25	<.001
Generated *sugar* function data count	0.03	.86

^a^HbA_1c_: hemoglobin A_1c_.

^b^DCSI: diabetes complications severity index.

### Comparison of Hemoglobin A_1c_ Regulation With Target Level in Continuous Use

Table 3 lists the proportion with regard to HbA_1c_ patterns. The proportion of users with the first HbA_1c_ measurement higher than 6.5% and the last HbA_1c_ measurement lower than 6.5% had a statistical difference (*P*=.01). Among users with the first HbA_1c_ measurement lower than 6.5%, the proportion of patients with the last HbA_1c_ measurement lower than 6.5% and the last HbA_1c_ measurement higher than 6.5% had no significant difference (*P*=.34 and *P*=.29, respectively). No significant difference was found between proportions of patients with the first HbA_1c_ measurement of 6.5% or higher and the last HbA_1c_ measurement higher than 6.5% (*P*=.41).

Similar to the *decline* analysis, the result of confounder adjustment by logistic regression for users with a high first HbA_1c_ measurement is summarized in Table 4. The continuous use of MCMH version 2.0 had a statistically significant effect in helping users move from an HbA_1c_ measurement above 6.5% to an HbA_1c_ measurement below 6.5% (*P*=.04). In addition, age, first HbA_1c_ measurement, and HbA_1c_ measurement in weeks before MCMH version 2.0 start showed statistically significant effects (all: *P*<.001).

**Table 3:** Pre– and post–hemoglobin A_1c_ management comparison by continuous use.

HbA_1c_^a^ pattern			Users		*P* value
			Continuous (n=133)	Noncontinuous (n=7320)	
**First measurement <6.5%**					
	**Last measurement**				
		<6.5%, n (%)	15 (11.3)	1040 (14.21)	.34
		≥6.5%, n (%)	7 (5.3)	564 (7.70)	.29
**First measurement** **≥** **6.5%**					
	**Last measurement**				
		<6.5%, n (%)	38 (28.6)	564 (7.70)	.01
		≥6.5%, n (%)	73 (54.9)	4278 (58.44)	.41

^a^HbA_1c_: hemoglobin A_1c_.

**Table 4:** The result of logistic regression against users with a high first hemoglobin A_1c_ measurement (n=111 continuous and n=5716 noncontinuous users).

Variables	Coefficient	*P* value
Constant	1.640	<.001
Continuous	0.618	.04
Age (years)	−0.010	<.001
Sex	−0.085	.20
First HbA_1c_^a^ measurement	−0.171	<.001
DCSI^b^	−0.041	.05
*Sugar* function data generation (weeks)	−0.004	.23
HbA_1c_ measurement in weeks before MCMH^c^ version 2.0 use start	−0.008	<.001
Generated *sugar* function data count	−0.001	.52

^a^HbA_1c_: hemoglobin A_1c_.

^b^DCSI: diabetes complications severity index.

^c^MCMH: My Chart in My Hand.

## Discussion

### Principal Findings

For the following reasons, this study supports the use of mPHRs as an effective platform for diabetes management by integrating patient-generated health and clinical data from PHRs and EMRs, respectively. First, analyzing the characteristics of continuous users of MCMH version 2.0, male patients with a high HbA_1c_ level seemed to use MCMH version 2.0 more continuously. Second, the continuous use of PHRs resulted in a higher decrease of HbA_1c_ levels and enhanced the regulation of high HbA_1c_ levels of patients to the target range. Therefore, male users with high HbA_1c_ levels had a higher decrease in HbA_1c_ levels and improved HbA_1c_ regulation to the target level. By analyzing the characteristics of continuous users and their HbA_1c_ patterns, we also suggest the use of mPHR as a diabetes care support tool enabling personalized management.

This study is unique when compared with previous studies on the basis of the following characteristics. First, we suggested the health improvement effect of mPHRs on the basis of the integration of PHRs and EMRs. In this study, we expected two benefits of integrating PHRs and EMRs. One is suggesting a different methodology for real-world data analysis and presenting additional real-world evidence, which supports previous studies. Another is ensuring a high-quality data analysis is conducted. There are many previous studies implying the advantages of PHRs and PGHD with positive conclusions of the use of mPHRs [14-17]. The results of these studies were collected on the basis of clinical trials such as nonblinded, open-label randomized controlled trials (RCTs) and cluster-randomized trial designs. As a real-world data analysis covers bias limitations in RCTs and can handle unknown factors of PHRs, the results of a real-world data analysis provide strong and necessary support to previous RCTs [25]. Moreover, the integration of EMRs gave high-quality HbA_1c_ data and diagnosis data, which made the analysis more precise.

Second, previous studies mainly discussed about the decrease in HbA_1c_ levels as an advantage of using PHRs. However, as the main goal of glycemic control is regulating a patient’s HbA_1c_ level to the recommended range, we compared both HbA_1c_ decrease and proportions of patients who initially had a high HbA_1c_ level but their HbA_1c_ level decreased to a low value. According to the 2015 and 2019 diabetes management guidelines from the KDA, the recommended target HbA_1c_ level is 6.5% in patients with type 2 diabetes, and this differs from the guideline by the ADA [4,26,27]. As this study was conducted in AMC, South Korea, we used the guidelines from KDA and defined the cutoff value of the HbA_1c_ level as 6.5%. Recent studies recommend that patients with severe diabetes mellitus should be controlled to lower than 7%, depending on the severity and complications of diabetes [28-30]. Moreover, a stable decrease in blood glucose levels is also an important task in glycemic control. We also focused on the *r*-squared value of the trend line and SD as an indicator of stabilized HbA_1c_ decrease, but we could not achieve any outstanding results.

### Overall User Characteristics

Analyzing users who had access to MCMH version 1.0 indicated that these users visited hospitals more with chronic diseases [21]. Continuous users were younger than noncontinuous users (*P*<.001), and there was a significant difference in sex proportion; the continuous user group had a higher male ratio (*P*<.001). In previous research, groups that used a PHR system had young users and a high proportion of males or generated more PGHD, especially those related to diabetes [21,22]. This is because male users aged between 51 and 70 years tend to adopt the PHR system [31]. In addition, in this study, the HbA_1c_ level in continuous users was measured for a shorter period (*P*=.04) and more frequently (*P*=.007) than noncontinuous users. However, the number of HbA_1c_ measurements had no significant difference between continuous and noncontinuous user groups. In South Korea, the social health insurance program was introduced with the 1977 National Health Insurance Act. This program was thereafter progressively rolled out to the general public, and it finally achieved universal coverage in 1989. According to the National Health Insurance Act, the criteria for the method, procedure, scope, and upper limit of health care shall be prescribed by the Ministry of Health and Welfare [17].

National insurance only supports up to 6 HbA_1c_ tests per year, in accordance with the National Health Insurance Act. First, we considered the number of HbA_1c_ measurements as another indicator of diabetes severity. This is because well-controlled patients typically undergo HbA_1c_ tests twice a year, whereas poorly controlled individuals undergo testing 4 times a year [32]. However, the number of measurements seems to be similar because of the policy in South Korea. Although continuous users had shorter periods (approximately 80 days) between the first and last measurements, this group took HbA_1c_ tests more frequently. This may be because of the increase in hospital visits, along with more satisfaction and loyalty to the hospital [33]. To compare diabetes severity, the proportion of patients with an HbA_1c_ level of 6.5% or above, a first HbA_1c_ level measurement, and a DCSI score distribution were compared between continuous and noncontinuous groups. The two groups had no significant difference in the proportion of high HbA_1c_ levels and DCSI distribution; however, continuous users had a higher HbA_1c_ level (*P*=.01). Retinopathy patients tended to use MCMH version 2.0 more continuously, but the complication proportion also had an insignificant difference between the two groups. Except for the first HbA_1c_ level measurement, most diabetic-related baseline characteristics appeared to have no significant difference, and the first HbA_1c_ measurement can be adjusted as confounders in an additional analysis. By using PHR and EMR integration, the general characteristics and severity of diabetes were compared.

As the period of HbA_1c_ measurement before MCMH version 2.0 use was shorter in the continuous group (*P*<.001), continuous users seemed to have an earlier MCMH version 2.0 start compared with noncontinuous users. In addition, continuous users tended to use the *sugar* and *diabetes calendar* functions more and generate more data. This was because continuous users tended to use MCMH version 2.0 functions with fewer burdens.

### Verifying the Effect of Personal Health Record Use in Hemoglobin A_1c_ Control

The main advantage of PHRs and PGHD is health improvement, especially in diabetes. Among the types of diabetes management, determining the change in HbA_1c_ levels was the most effective method to verify the effectiveness of PHRs in the real world. The results of this study indicate that continuous users had a larger *decline*; a greater increase in HbA_1c_ levels was observed in users who continuously used the diabetes management–related *sugar* function in MCMH version 2.0. As *decline* is the result of the trend line slope normalized to 100 days, the value itself also refers to the change in the HbA_1c_ level. For example, HbA_1c_ was 6.9% on January 1, 2014, and HbA_1c_ was 6.4% on October 19, 2018, in one particular continuous user; therefore, the decline value was −0.0044, which means that this patient’s change in HbA_1c_ level was approximately −0.44% (100 times the value of decline). Thus, the decrease in HbA_1c_ levels in continuous users was approximately 1.9 times that in noncontinuous users. The result of ANCOVA shows that along with continuous use, other factors were also important: age, first HbA_1c_ measurement, DCSI, duration of using the *sugar* function, and HbA_1c_ measurement period before using MCMH version 2.0. Glycemic control is important for reducing both microvascular risk and emergent risk for myocardial infarction and death [34]. This indicates that the group that continuously used PHRs had health improvement with a decreasing trend of HbA_1c_ levels.

In glycemic control, it is important to reduce not only blood glucose levels but also hypoglycemic events [35]. Traditional diabetes care includes insulin delivery using syringes, pens, or pumps [3]. Although hypoglycemic side effects can occur with multiple daily injections and continuous subcutaneous insulin injection, the invasive characteristic of such forms of care is an inevitable disadvantage [36-39]. In this study, we tried to minimize the risk of hypoglycemic events in PHR-implemented diabetes management by using stability indicators, *r*-squared value and *SD*. However, stability was not ensured. In fact, a previous study showed increased glucose stability with the use of an internet-based glucose monitoring system [40]. This indicates that patients can improve hyperglycemia and hypoglycemia management by using PHRs with a blood glucose meter through continuous glucose monitoring diabetic care.

The goal of decreasing the HbA_1c_ level is to prevent the occurrence and aggravation of diabetic complications. Although the criterion for HbA_1c_ in a diagnostic test for diabetes has been recommended by the American Association of Clinical Endocrinologists and ADA, it is an “acceptable complementary diagnostic test for diabetes in Korean patients” [28,41]. Among the many glycemic controls, the tight regulation of HbA_1c_ levels is essential for health improvement and for lowering complication risks such as diabetic retinopathy [42]. In addition, the tight glycemic control of HbA_1c_ levels to 7.0% induces a lower risk of fracture in elderly patients with diabetes [43]. When comparing the ratio of patients with HbA_1c_ levels above and below 6.5% before and after the use of MCMH version 2.0, the group that continuously used MCMH version 2.0 had a higher proportion of regulated patients; initially, the first HbA_1c_ level measurement was over 6.5%, and then it reduced to lower than 6.5%. In addition, among users with the first HbA_1c_ level measurement over 6.5%, the logistic regression results showed that regulation was associated not only with continuous use but also with age, first HbA_1c_ level measurement, and how fast MCMH version 2.0 was adapted. The data generation amount was thought to be important too, but it was statistically insignificant. Therefore, we can claim that the improvement of HbA_1c_ levels by PHR use can eventually affect diabetes management by controlling HbA_1c_ levels to 6.5% in practice.

### Limitations of This Research

The main limitation of this study is the concern of general biases in real-world studies: selection bias, information bias, recall bias, and detection bias [44]. As this study mainly focused on analyzing real-world data, strict criteria and inevitable exclusion are necessary, leading to concerns in selection bias and detection bias. However, the criteria for the comparison group were the same, and despite including and excluding many patient criteria and comparing with the MCMH 1.0 user analysis, the study scale is almost similar [22]. The size of the continuous user groups is sometimes larger than that used in other RCT studies and had little baseline differences in diabetic severity [17]. As MCMH version 2.0 data are PGHD, continuous use can only be analyzed by its log data, which does not represent adherence to the app and can lead to information bias. On the contrary, we note that information bias that can occur in HbA_1c_ level scaling can be controlled with the integration of EMRs. This integration helped in reducing recall bias in diabetes and complication diagnosis.

Time scale is also another limitation. In RCTs, the HbA_1c_ measurement point, the app account creation point, and app use frequency can be controlled and optimized for convenient data analysis. However, in real-world data, patients have diverse points of HbA_1c_ measurement and MCMH version 2.0 starting points. Even though there were limitations with regard to missing data, inappropriate data, and ambiguous time scale standards, we used patient selection criteria to choose patients who can be analyzed and used the *decline* factor to monitor the HbA_1c_ level for minimizing the effect of irregular time points. The *decline* factor is a variable that has been coined for the purpose of this study and has an uncertain clinical rationale. However, as the *decline* variable also implies a decrease in HbA_1c_ levels, and the decreasing trend is being maintained, the quantitative comparison of *decline* between groups is meaningful. In diabetes care, lowering HbA_1c_ levels to the target level and maintaining the decreased HbA_1c_ level is the primary goal. Thus, the *decline* is a reasonable variable for analysis in studies with data having unspecific HbA_1c_ measurement points.

An additional limitation is that AMC is a territorial hospital, and almost all the study patients are residing in South Korea. The small size of the study population and short duration are other limitations. The low frequency of PHR data generation and short-term MCMH version 2.0 operation is not an ideal database for analyzing chronic diseases such as diabetes. A larger study size and longer study duration will provide stronger real-world evidence of the clinical meaning of PHRs.

On the basis of the proportion of continuous and noncontinuous users, further research for encouraging patients to use PHRs more continuously is essential. In this study, continuous users had better diabetes management outcomes than noncontinuous users. However, continuous users were only 1.78% (133/7453) of the study population and were only 0.20% (133/64,932) of users who started using MCMH version 2.0. Thus, studies for maintaining active PGHD-generating users and turning noncontinuous users into continuous users are necessary. Finding out whether giving health-related advice on the basis of MCMH version 2.0 encourages patients to use a PHR app for changing app use patterns needs to be studied to prevent usability issues [45]. Furthermore, for personalized PHR advice, if larger and better quality of data is provided, the glycemic control outcome analysis by treatment is important. Further studies in diverse territories and a deeper analysis of MCMH version 2.0 should be performed to prove the effectiveness of PHRs as a diabetes management tool in decreasing HbA_1c_ levels.

### Conclusions

By integrating and analyzing patient- and clinically generated data, the continuous use of PHRs improves diabetes management outcomes. A greater decrease in HbA_1c_ levels was observed in continuous users, and HbA_1c_ levels were regulated to the target level in continuous users compared with noncontinuous users. Previous clinical trials and the results of this study proved that PHRs are effective in managing diabetes. However, further evaluation of the effectiveness of PHRs in various diseases and studies for adherence to PHRs are needed. A larger study population and longer duration will be necessary for the accurate analysis of the clinical rationale of PHRs on chronic diseases.

## Appendix

Multimedia Appendix 1 Formula of decline.

Multimedia Appendix 2 Diabetes complications severity index score proportion comparison of continuous and noncontinuous users.

## Abbreviations

ADA: American Diabetes Association
AMC: Asan Medical Center
ANCOVA: analysis of covariance
DCSI: diabetes complications severity index
EMR: electronic medical record
HbA_1c_: hemoglobin A_1c_
ICD-10: International Classification of Diseases 10th Revision
IRB: institutional review board
KDA: Korean Diabetes Association
MCMH: My Chart in My Hand
mHealth: mobile health
mPHR: mobile personal health record
PGHD: patient-generated health data
PHR: personal health record
RCT: randomized controlled trial
