# Supplementary figures and images for: The Use of Mobile Personal Health Records for Hemoglobin A1c Regulation in Patients With Diabetes: Retrospective Observational Study
Source: J Med Internet Res. 2020 Jun 2;22(6):e15372. doi: 10.2196/15372 (PMC7298631; doi:10.2196/15372)

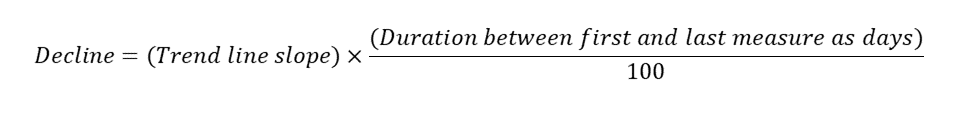

Supplement: Multimedia Appendix 1 [file jmir_v22i6e15372_app1.png]

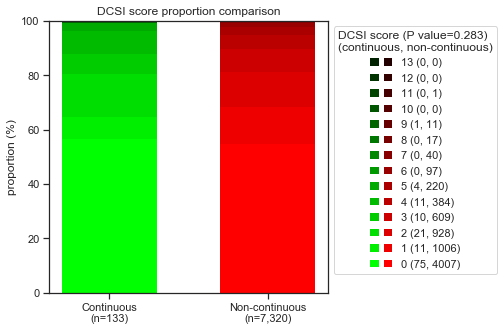

Supplement: Multimedia Appendix 2 [file jmir_v22i6e15372_app2.png]
